# Supplementary figures and images for: Cellular reprogramming with ATOH1, GFI1, and POU4F3 implicate epigenetic changes and cell-cell signaling as obstacles to hair cell regeneration in mature mammals
Source: eLife. 2022 Nov 29;11:e79712. doi: 10.7554/eLife.79712 (PMC9708077; doi:10.7554/eLife.79712)

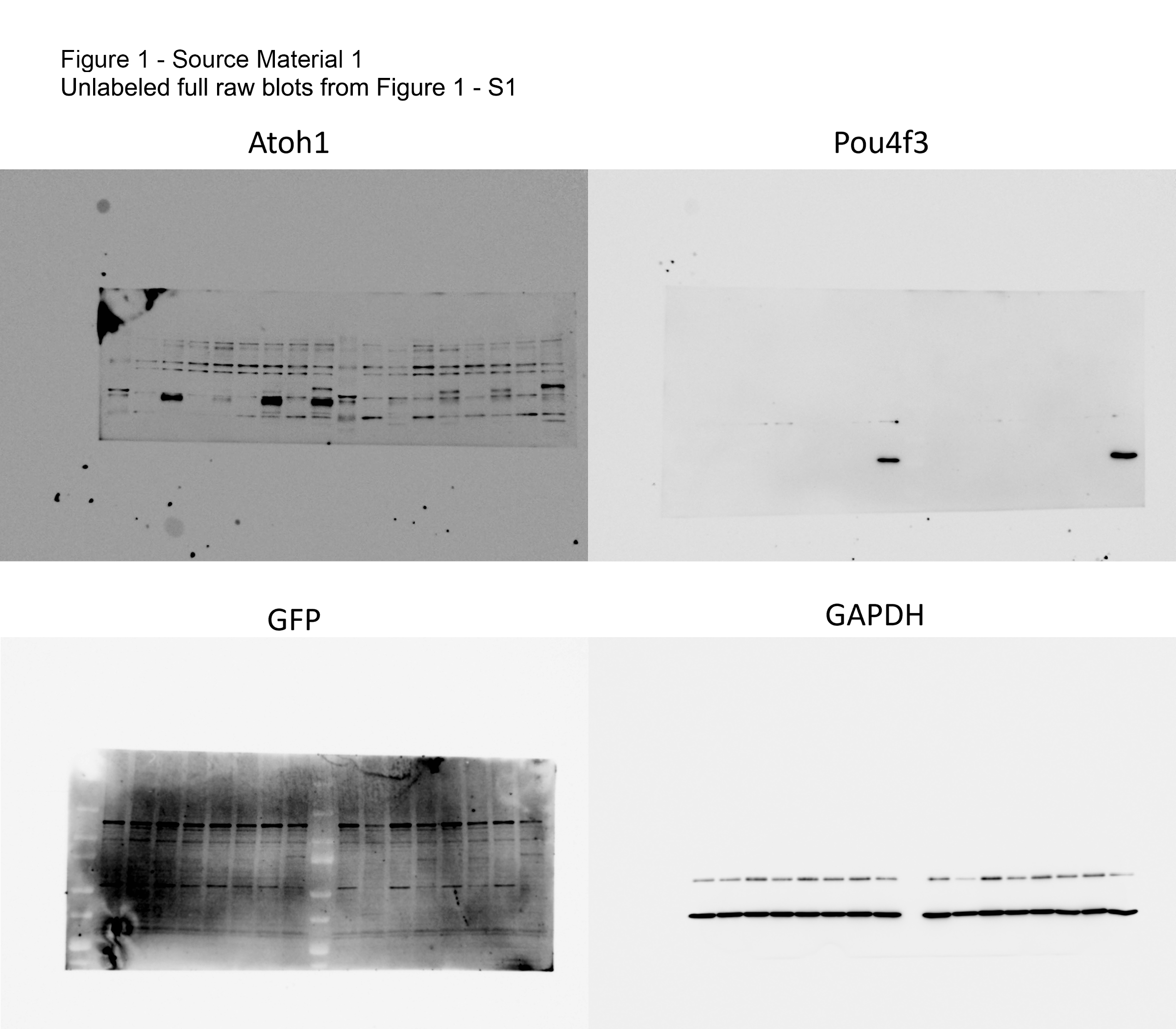

Supplement: Figure 1—source data 1. — Western blotting was performed after 48h with antibodies specific to ATOH1, GFP, POU4F3 , and GAPDH as a loading control. The raw blots are shown. [file elife-79712-fig1-data1.zip › Figure 1 Source Data 1.tif]

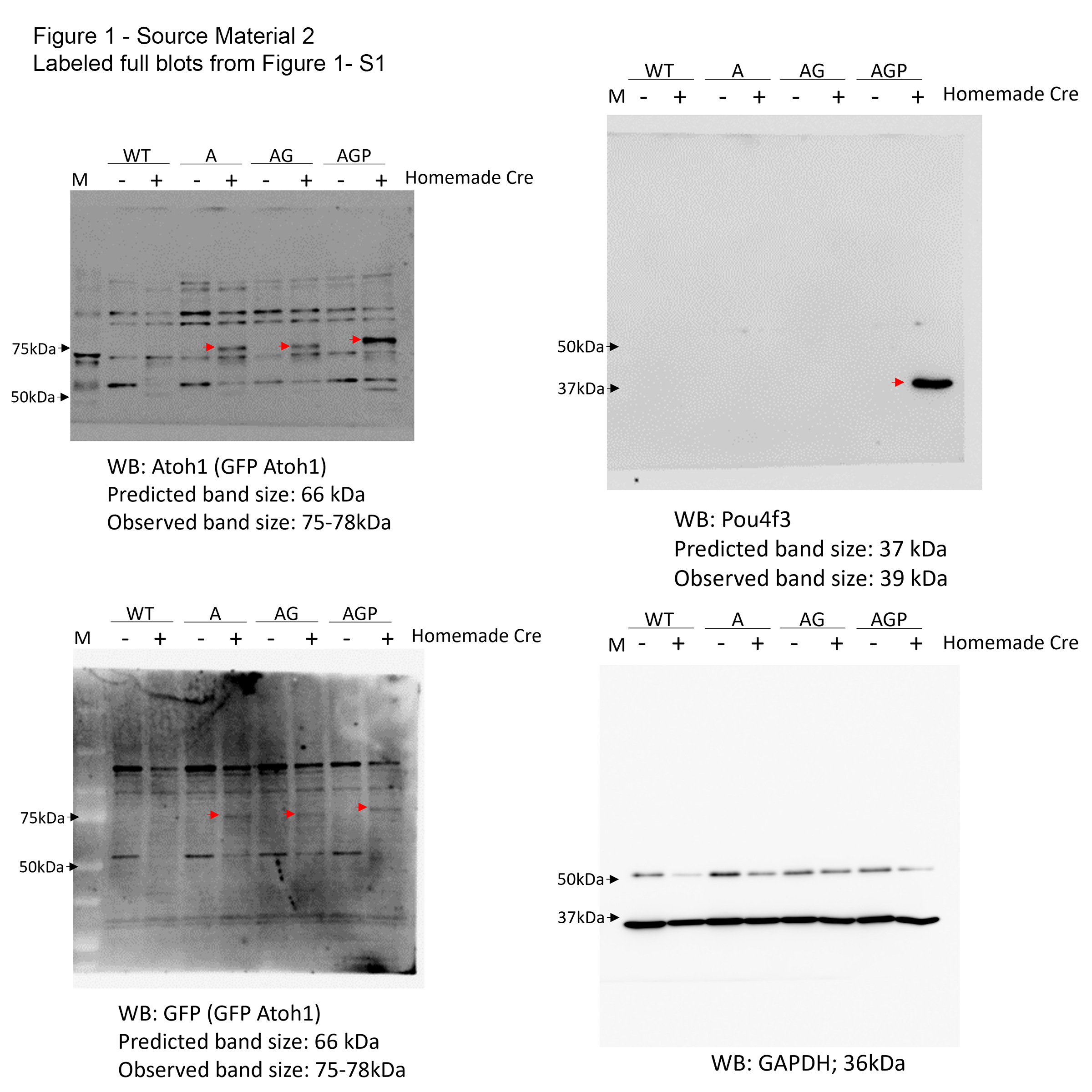

Supplement: Figure 1—source data 2. — Western blotting was performed after 48h with antibodies specific to ATOH1, GFP, POU4F3 , and GAPDH as a loading control. The raw blots are shown with labels attached to indicate the relevant bands and the conditions used. [file elife-79712-fig1-data2.zip › Figure 1 Source Data 2.tif]
