# Supplementary material for: Cellular reprogramming with ATOH1, GFI1, and POU4F3 implicate epigenetic changes and cell-cell signaling as obstacles to hair cell regeneration in mature mammals
Source: eLife. 2022 Nov 29;11:e79712. doi: 10.7554/eLife.79712 (PMC9708077; doi:10.7554/eLife.79712)
Supplement: Figure 8—source data 1. [file elife-79712-fig8-data1.docx]

**Figure 8 – source data 1** - List of genes downregulated in supporting cells in response to transcription factor reprogramming at P15

| **Gene** | **Structural/Functional role** | **Expression** | **Reference** |
| --- | --- | --- | --- |
| *Ceacam16* | Enables polymerization of tectorins to form the tectorial membrane | SC (Deiters’ cells), Interdental cells | [(Kammerer et al., 2012)](https://paperpile.com/c/06Otv1/0f9pn) |
| *Ttll3* | Microtubule based process, cytoskeleton | SC (Deiters’ cells) | [(Huang et al., 2011; Ranum et al., 2019)](https://paperpile.com/c/06Otv1/RVlb+mjSe) |
| *Rorb* | Transcription factor required for terminal SC differentiation | SC (Deiters’ and pillar cells) | [(Li et al., 2020b)](https://paperpile.com/c/06Otv1/JBBB) |
| *Scd1* | Lipogenic enzyme essential for phospholipid synthesis | SC (Deiters’ cells) | [(Ranum et al., 2019)](https://paperpile.com/c/06Otv1/RVlb) |
| *Rassf6* | Involved in cell death and apoptosis | SC (Pillar cells) | [(Maass et al., 2016)](https://paperpile.com/c/06Otv1/Zo7Y) |
| *Ces1d* | Carboxylesterase 1D. Enables triglyceride lipase activity | SC (Deiters’ cells) | [(Ranum et al., 2019)](https://paperpile.com/c/06Otv1/RVlb) |
| *Fibin* | Secreted protein for mesoderm signaling during development | GER, SC (inner phalangeal cell marker) | [(Kolla et al., 2020; Wakahara et al., 2007)](https://paperpile.com/c/06Otv1/VRNY+WCox) |
| *Scnn1b* | Ion channel, membrane transporter | SC(Claudius cells), Reissner’s membrane | [(Li et al., 2013)](https://paperpile.com/c/06Otv1/tNAc) |
| *Kcnj16* | K+ channel involved in endocochlear potential maintenance | SC (Deiters cells) | [(Liu et al., 2018)](https://paperpile.com/c/06Otv1/xw2Y) |
| *Otol1* | Provides a scaffold for otoconia, anchoring protein | SC (Pillar cells) | [(Liu et al., 2018; Waldhaus et al., 2015)](https://paperpile.com/c/06Otv1/8COO+xw2Y) |
| *Plcd4* | Non-receptor based G-protein activator enzyme | SC (Pillar & Deiters cells) | [(Liu et al., 2018)](https://paperpile.com/c/06Otv1/xw2Y) |
| *Hhatl* | Codes for Hedgehog acyl transferase | SC (Pillar & Deiters cells) | [(Liu et al., 2018)](https://paperpile.com/c/06Otv1/xw2Y) |
| *Washc2* | Part of WASH complex, nucleation promoting factor on endosomes | SC (minimal expression in Pillar & Deiters cells) | [(Liu et al., 2018)](https://paperpile.com/c/06Otv1/xw2Y) |
| *Hbb-bs* | Hemoglobin beta chain, blood cell marker | Cochlear vasculature | [(Li et al., 2020a)](https://paperpile.com/c/06Otv1/TaXQ) |

**References**

[Huang M, Sage C, Tang Y, Lee SG, Petrillo M, Hinds PW, Chen Z-Y. 2011. Overlapping and distinct pRb pathways in the mammalian auditory and vestibular organs. *Cell Cycle* **10**:337–351. doi:](http://paperpile.com/b/06Otv1/mjSe)[10.4161/cc.10.2.14640](http://dx.doi.org/10.4161/cc.10.2.14640)

[Kammerer R, Rüttiger L, Riesenberg R, Schäuble C, Krupar R, Kamp A, Sunami K, Eisenried A, Hennenberg M, Grunert F, Bress A, Battaglia S, Schrewe H, Knipper M, Schneider MR, Zimmermann W. 2012. Loss of Mammal-specific Tectorial Membrane Component Carcinoembryonic Antigen Cell Adhesion Molecule 16 (CEACAM16) Leads to Hearing Impairment at Low and High Frequencies. *Journal of Biological Chemistry*. doi:](http://paperpile.com/b/06Otv1/0f9pn)[10.1074/jbc.m111.320481](http://dx.doi.org/10.1074/jbc.m111.320481)

[Kolla L, Kelly MC, Mann ZF, Anaya-Rocha A, Ellis K, Lemons A, Palermo AT, So KS, Mays JC, Orvis J, Burns JC, Hertzano R, Driver EC, Kelley MW. 2020. Characterization of the development of the mouse cochlear epithelium at the single cell level. *Nat Commun* **11**:2389. doi:](http://paperpile.com/b/06Otv1/VRNY)[10.1038/s41467-020-16113-y](http://dx.doi.org/10.1038/s41467-020-16113-y)

[Li C, Li X, Bi Z, Sugino K, Wang G, Zhu T, Liu Z. 2020a. Comprehensive transcriptome analysis of cochlear spiral ganglion neurons at multiple ages. *Elife* **9**. doi:](http://paperpile.com/b/06Otv1/TaXQ)[10.7554/eLife.50491](http://dx.doi.org/10.7554/eLife.50491)

[Li C, Wang Y, Wang G, Lu Y, He S, Sun Y, Liu Z. 2020b. Fate-mapping analysis using Rorb-IRES-Cre reveals apical-to-basal gradient of Rorb expression in mouse cochlea. *Dev Dyn* **249**:173–186. doi:](http://paperpile.com/b/06Otv1/JBBB)[10.1002/dvdy.111](http://dx.doi.org/10.1002/dvdy.111)

[Liu H, Chen L, Giffen KP, Stringham ST, Li Y, Judge PD, Beisel KW, He DZZ. 2018. Cell-Specific Transcriptome Analysis Shows That Adult Pillar and Deiters’ Cells Express Genes Encoding Machinery for Specializations of Cochlear Hair Cells. *Frontiers in Molecular Neuroscience*. doi:](http://paperpile.com/b/06Otv1/xw2Y)[10.3389/fnmol.2018.00356](http://dx.doi.org/10.3389/fnmol.2018.00356)

[Li X, Zhou F, Marcus DC, Wangemann P. 2013. Endolymphatic Na+ and K+ Concentrations during Cochlear Growth and Enlargement in Mice Lacking Slc26a4/pendrin. *PLoS One* **8**:e65977. doi:](http://paperpile.com/b/06Otv1/tNAc)[10.1371/journal.pone.0065977](http://dx.doi.org/10.1371/journal.pone.0065977)

[Maass JC, Gu R, Cai T, Wan Y-W, Cantellano SC, Asprer JST, Zhang H, Jen H-I, Edlund RK, Liu Z, Groves AK. 2016. Transcriptomic Analysis of Mouse Cochlear Supporting Cell Maturation Reveals Large-Scale Changes in Notch Responsiveness Prior to the Onset of Hearing. *PLoS One* **11**:e0167286. doi:](http://paperpile.com/b/06Otv1/Zo7Y)[10.1371/journal.pone.0167286](http://dx.doi.org/10.1371/journal.pone.0167286)

[Ranum PT, Goodwin AT, Yoshimura H, Kolbe DL, Walls WD, Koh J-Y, He DZZ, Smith RJH. 2019. Insights into the Biology of Hearing and Deafness Revealed by Single-Cell RNA Sequencing. *Cell Rep* **26**:3160–3171.e3. doi:](http://paperpile.com/b/06Otv1/RVlb)[10.1016/j.celrep.2019.02.053](http://dx.doi.org/10.1016/j.celrep.2019.02.053)

[Wakahara T, Kusu N, Yamauchi H, Kimura I, Konishi M, Miyake A, Itoh N. 2007. Fibin, a novel secreted lateral plate mesoderm signal, is essential for pectoral fin bud initiation in zebrafish. *Dev Biol* **303**:527–535. doi:](http://paperpile.com/b/06Otv1/WCox)[10.1016/j.ydbio.2006.11.041](http://dx.doi.org/10.1016/j.ydbio.2006.11.041)

[Waldhaus J, Durruthy-Durruthy R, Heller S. 2015. Quantitative High-Resolution Cellular Map of the Organ of Corti. *Cell Rep* **11**:1385–1399. doi:](http://paperpile.com/b/06Otv1/8COO)[10.1016/j.celrep.2015.04.062](http://dx.doi.org/10.1016/j.celrep.2015.04.062)
