# Supplementary material for: Cellular reprogramming with ATOH1, GFI1, and POU4F3 implicate epigenetic changes and cell-cell signaling as obstacles to hair cell regeneration in mature mammals
Source: eLife. 2022 Nov 29;11:e79712. doi: 10.7554/eLife.79712 (PMC9708077; doi:10.7554/eLife.79712)
Supplement: Figure 8—source data 2. [file elife-79712-fig8-data2.docx]

**Figure 8 – source data 2** - List of genes upregulated in supporting cells in response to transcription factor reprogramming at P15

| **Gene** | **Structural/Functional role** | **Expression** | **Reference** |
| --- | --- | --- | --- |
| *Cib2* | Binds to TMC1 and TMC2 for normal function of mechanotransduction channels | Hair cells | [(Giese et al., 2017)](https://paperpile.com/c/WgJNfa/Qcte) |
| *Hes6* | ATOH1 target gene which delineates sensory lineage in the inner ear. | Hair cells | [(Qian et al., 2006)](https://paperpile.com/c/WgJNfa/Xry4) |
| *Igfbp6* | Glycoprotein | Hair cells | [(Scheffer et al., 2015)](https://paperpile.com/c/WgJNfa/QqKK) |
| *Krt8* | Epithelial gene induced in response to epigenetic DNA methylation in sensory epithelia derived progenitors | Induced hair cells | [(Zhou and Hu, 2015)](https://paperpile.com/c/WgJNfa/3pr2) |
| *Mfng* | Co-expressed with *Atoh1* in hair cells | Hair cells | [(Basch et al., 2016)](https://paperpile.com/c/WgJNfa/MDyR) |
| *Mgst3* | Catalyzes oxidation of hydroxy-fatty acids. Role in lipid metabolism. | Hair cells | [(Scheffer et al., 2015)](https://paperpile.com/c/WgJNfa/QqKK) |
| *Otoa* | Links hair cells to acellular gels connecting them to the tectorial membrane | Transient- Hair cells | [(Zwaenepoel et al., 2002)](https://paperpile.com/c/WgJNfa/y7hS) |
| *Acbd7* | Protein involved in lipid binding and Fatty-Acyl-CoA biosynthesis pathway | Hair cells | [(Kolla et al., 2020; Scheffer et al., 2015)](https://paperpile.com/c/WgJNfa/sdoj+QqKK) |
| *Calb2* | Calcium binding protein which regulates calcium influx during sound transduction. | Hair cells and SGN | [(Liu et al., 2016)](https://paperpile.com/c/WgJNfa/QqQR) |
| *Calml4* | Calmodulin 4 which functions as a myosin light chain component. Expressed along with *Atoh1* | Hair cells | [(Scheffer et al., 2015; Yoon et al., 2011)](https://paperpile.com/c/WgJNfa/QqKK+l6aG) |
| *Ccer2* | Coiled-Coil Glutamate Rich Protein 2. Unknown significance. | Hair cells | [(Kolla et al., 2020)](https://paperpile.com/c/WgJNfa/sdoj) |
| *Agr3* | ATOH1 target gene. Plays a role in regulation of cilia | Hair cells | [(Yoon et al., 2011)](https://paperpile.com/c/WgJNfa/l6aG) |
| *Dlk2* | DLK2 (Delta Like Non-Canonical Notch Ligand 2) plays a role in the Notch signaling | Hair cells | [(Cai et al., 2015; Scheffer et al., 2015)](https://paperpile.com/c/WgJNfa/QqKK+67A9) |
| *Lmo1* | Transcription factor | Hair cells | [(Deng et al., 2006)](https://paperpile.com/c/WgJNfa/2FyL) |
| *Myl9* | Myosin light chain protein involved in hair cell shape changes | Hair cells | [(Oya et al., 2021)](https://paperpile.com/c/WgJNfa/92dO) |
| *Tmem255b* | Transmembrane protein upregulated with Notch inhibition | Hair cell | Angelika Doetzlhofer lab (Paul Campbell, thesis) |
| *Col2a1* | Pro collagen protein. Mutations in Col2a1 lead to auditory defects (Stickler syndrome) | Hair Cells | [(Khetarpal et al., 1994)](https://paperpile.com/c/WgJNfa/vDrE) |
| *Slc5a3* | Participates in osmoregulation, required to form synaptic terminals | Hair cells (vestibular) | [(Scheffer et al., 2015)](https://paperpile.com/c/WgJNfa/QqKK) |
| *Tubb2b* | Interacts with ATOH1. Tubulin component of microtubules. | Hair cells | [(Cai et al., 2015; Chessum et al., 2018)](https://paperpile.com/c/WgJNfa/jLMU+67A9) |
| *Fstl1* | BMP4 signaling antagonist. BMP signaling inhibition known to increase sensory patch and HC differentiation | Hair cells | [(Cai et al., 2015)](https://paperpile.com/c/WgJNfa/67A9) |
| *Itga6* | CD49F known to increase multipotency of cells through induction of OCT-4 and SOX-2 | Hair cells | [(Cai et al., 2015)](https://paperpile.com/c/WgJNfa/67A9) |
| *Fabp5* | Protein which plays a role in fatty acid uptake, transport, and metabolism | Hair cells | [(Cai et al., 2015; Chessum et al., 2018; Liu et al., 2018)](https://paperpile.com/c/WgJNfa/67A9+jLMU+8UfP) |
| *Gm266* | GTPase activity, GDP binding protein, involved in signal transduction | Hair cells | [(Cai et al., 2015; Chessum et al., 2018; Liu et al., 2018)](https://paperpile.com/c/WgJNfa/67A9+jLMU+8UfP) |
| *Miat* | Non protein coding transcript | Hair cells | [(Kolla et al., 2020; Liu et al., 2018)](https://paperpile.com/c/WgJNfa/sdoj+8UfP) |
| *Ccnd1* | Cyclin D1 expression in supporting cells correlates to its proliferative capacity | Early postnatal HC and proliferative SC | [(Laine et al., 2010)](https://paperpile.com/c/WgJNfa/wcGr) |
| *Mdk* | Neurotrophic factor, activates *Notch2* | SC (Vestibular),  Notch pathway | [(Chessum et al., 2018)](https://paperpile.com/c/WgJNfa/jLMU) |
| *Hes5* | Downregulated in postnatal cochlea and along with Notch is known to accelerate regeneration potential in neonates and vestibular system | SC, Notch pathway | [(Kubota et al., 2021)](https://paperpile.com/c/WgJNfa/DAqE) |
| *Uchl1* | Increases expression of beta-catenin and regulates wnt signaling | SC, Wnt signaling pathway | [(Chessum et al., 2018; Kolla et al., 2020)](https://paperpile.com/c/WgJNfa/jLMU+sdoj) |
| *Igfbp3* | Glycoprotein. Prosensory cell marker whose expression is regulated by *Jag1* during development. | SC (Inner phalangeal/ border, Deiters’ cells),  Notch pathway | [(Chrysostomou et al., 2020; Okano and Kelley, 2013)](https://paperpile.com/c/WgJNfa/w9HV+hea0) |

**References**

[Basch ML, Brown RM 2nd, Jen H-I, Semerci F, Depreux F, Edlund RK, Zhang H, Norton CR, Gridley T, Cole SE, Doetzlhofer A, Maletic-Savatic M, Segil N, Groves AK. 2016. Fine-tuning of Notch signaling sets the boundary of the organ of Corti and establishes sensory cell fates. *Elife* **5**. doi:](http://paperpile.com/b/WgJNfa/MDyR)[10.7554/eLife.19921](http://dx.doi.org/10.7554/eLife.19921)

[Cai T, Jen H-I, Kang H, Klisch TJ, Zoghbi HY, Groves AK. 2015. Characterization of the transcriptome of nascent hair cells and identification of direct targets of the Atoh1 transcription factor. *J Neurosci* **35**:5870–5883. doi:](http://paperpile.com/b/WgJNfa/67A9)[10.1523/JNEUROSCI.5083-14.2015](http://dx.doi.org/10.1523/JNEUROSCI.5083-14.2015)

[Chessum L, Matern MS, Kelly MC, Johnson SL, Ogawa Y, Milon B, McMurray M, Driver EC, Parker A, Song Y, Codner G, Esapa CT, Prescott J, Trent G, Wells S, Dragich AK, Frolenkov GI, Kelley MW, Marcotti W, Brown SDM, Elkon R, Bowl MR, Hertzano R. 2018. Helios is a key transcriptional regulator of outer hair cell maturation. *Nature* **563**:696–700. doi:](http://paperpile.com/b/WgJNfa/jLMU)[10.1038/s41586-018-0728-4](http://dx.doi.org/10.1038/s41586-018-0728-4)

[Chrysostomou E, Zhou L, Darcy YL, Graves KA, Doetzlhofer A, Cox BC. 2020. The Notch Ligand Jagged1 Is Required for the Formation, Maintenance, and Survival of Hensen’s Cells in the Mouse Cochlea. *J Neurosci* **40**:9401–9413. doi:](http://paperpile.com/b/WgJNfa/w9HV)[10.1523/JNEUROSCI.1192-20.2020](http://dx.doi.org/10.1523/JNEUROSCI.1192-20.2020)

[Deng M, Pan L, Xie X, Gan L. 2006. Differential expression of LIM domain-only (LMO) genes in the developing mouse inner ear. *Gene Expr Patterns* **6**:857–863. doi:](http://paperpile.com/b/WgJNfa/2FyL)[10.1016/j.modgep.2006.02.005](http://dx.doi.org/10.1016/j.modgep.2006.02.005)

[Giese APJ, Tang Y-Q, Sinha GP, Bowl MR, Goldring AC, Parker A, Freeman MJ, Brown SDM, Riazuddin S, Fettiplace R, Schafer WR, Frolenkov GI, Ahmed ZM. 2017. CIB2 interacts with TMC1 and TMC2 and is essential for mechanotransduction in auditory hair cells. *Nat Commun* **8**:43. doi:](http://paperpile.com/b/WgJNfa/Qcte)[10.1038/s41467-017-00061-1](http://dx.doi.org/10.1038/s41467-017-00061-1)

[Khetarpal U, Robertson NG, Yoo TJ, Morton CC. 1994. Expression and localization of COL2A1 mRNA and type II collagen in human fetal cochlea. *Hear Res* **79**:59–73. doi:](http://paperpile.com/b/WgJNfa/vDrE)[10.1016/0378-5955(94)90127-9](http://dx.doi.org/10.1016/0378-5955(94)90127-9)

[Kolla L, Kelly MC, Mann ZF, Anaya-Rocha A, Ellis K, Lemons A, Palermo AT, So KS, Mays JC, Orvis J, Burns JC, Hertzano R, Driver EC, Kelley MW. 2020. Characterization of the development of the mouse cochlear epithelium at the single cell level. *Nat Commun* **11**:2389. doi:](http://paperpile.com/b/WgJNfa/sdoj)[10.1038/s41467-020-16113-y](http://dx.doi.org/10.1038/s41467-020-16113-y)

[Kubota M, Scheibinger M, Jan TA, Heller S. 2021. Greater epithelial ridge cells are the principal organoid-forming progenitors of the mouse cochlea. *Cell Rep* **34**:108646. doi:](http://paperpile.com/b/WgJNfa/DAqE)[10.1016/j.celrep.2020.108646](http://dx.doi.org/10.1016/j.celrep.2020.108646)

[Laine H, Sulg M, Kirjavainen A, Pirvola U. 2010. Cell cycle regulation in the inner ear sensory epithelia: role of cyclin D1 and cyclin-dependent kinase inhibitors. *Dev Biol* **337**:134–146. doi:](http://paperpile.com/b/WgJNfa/wcGr)[10.1016/j.ydbio.2009.10.027](http://dx.doi.org/10.1016/j.ydbio.2009.10.027)

[Liu H, Chen L, Giffen KP, Stringham ST, Li Y, Judge PD, Beisel KW, He DZZ. 2018. Cell-Specific Transcriptome Analysis Shows That Adult Pillar and Deiters’ Cells Express Genes Encoding Machinery for Specializations of Cochlear Hair Cells. *Frontiers in Molecular Neuroscience*. doi:](http://paperpile.com/b/WgJNfa/8UfP)[10.3389/fnmol.2018.00356](http://dx.doi.org/10.3389/fnmol.2018.00356)

[Liu X-P, Koehler KR, Mikosz AM, Hashino E, Holt JR. 2016. Functional development of mechanosensitive hair cells in stem cell-derived organoids parallels native vestibular hair cells. *Nat Commun* **7**:11508. doi:](http://paperpile.com/b/WgJNfa/QqQR)[10.1038/ncomms11508](http://dx.doi.org/10.1038/ncomms11508)

[Okano T, Kelley MW. 2013. Expression of insulin-like growth factor binding proteins during mouse cochlear development. *Developmental Dynamics*. doi:](http://paperpile.com/b/WgJNfa/hea0)[10.1002/dvdy.24005](http://dx.doi.org/10.1002/dvdy.24005)

[Oya R, Tsukamoto O, Sato T, Kato H, Matsuoka K, Oshima K, Kamakura T, Ohta Y, Imai T, Takashima S, Inohara H. 2021. Phosphorylation of MYL12 by Myosin Light Chain Kinase Regulates Cellular Shape Changes in Cochlear Hair Cells. *Journal of the Association for Research in Otolaryngology*. doi:](http://paperpile.com/b/WgJNfa/92dO)[10.1007/s10162-021-00796-1](http://dx.doi.org/10.1007/s10162-021-00796-1)

[Qian D, Radde-Gallwitz K, Kelly M, Tyrberg B, Kim J, Gao W-Q, Chen P. 2006. Basic helix-loop-helix gene Hes6 delineates the sensory hair cell lineage in the inner ear. *Dev Dyn* **235**:1689–1700. doi:](http://paperpile.com/b/WgJNfa/Xry4)[10.1002/dvdy.20736](http://dx.doi.org/10.1002/dvdy.20736)

[Scheffer DI, Shen J, Corey DP, Chen Z-Y. 2015. Gene Expression by Mouse Inner Ear Hair Cells during Development. *J Neurosci* **35**:6366–6380. doi:](http://paperpile.com/b/WgJNfa/QqKK)[10.1523/JNEUROSCI.5126-14.2015](http://dx.doi.org/10.1523/JNEUROSCI.5126-14.2015)

[Yoon H, Lee DJ, Kim MH, Bok J. 2011. Identification of genes concordantly expressed with Atoh1 during inner ear development. *Anat Cell Biol* **44**:69–78. doi:](http://paperpile.com/b/WgJNfa/l6aG)[10.5115/acb.2011.44.1.69](http://dx.doi.org/10.5115/acb.2011.44.1.69)

[Zhou Y, Hu Z. 2015. Genome-wide demethylation by 5-aza-2’-deoxycytidine alters the cell fate of stem/progenitor cells. *Stem Cell Rev Rep* **11**:87–95. doi:](http://paperpile.com/b/WgJNfa/3pr2)[10.1007/s12015-014-9542-z](http://dx.doi.org/10.1007/s12015-014-9542-z)

[Zwaenepoel I, Mustapha M, Leibovici M, Verpy E, Goodyear R, Liu XZ, Nouaille S, Nance WE, Kanaan M, Avraham KB, Tekaia F, Loiselet J, Lathrop M, Richardson G, Petit C. 2002. Otoancorin, an inner ear protein restricted to the interface between the apical surface of sensory epithelia and their overlying acellular gels, is defective in autosomal recessive deafness DFNB22. *Proceedings of the National Academy of Sciences*. doi:](http://paperpile.com/b/WgJNfa/y7hS)[10.1073/pnas.082515999](http://dx.doi.org/10.1073/pnas.082515999)
